# Cases Treated with Veratrum Viride

**Published:** 1852-07

**Authors:** D. C. O’Keeffe


### SELECTIONS

FROM

### FOREIGN AND HOME MEDICAL JOURNALS.

---

*Cases treated with Veratrum Viride.* By Dr. D. C. O'Keeffe. (Reported by J. S. Clements, of Penfield, Ga.)

Case I. Nov. 3, 1851.—Mrs. ——, aged 21, was delivered of her first child one week ago; progressed satisfactorily until above date. Condition at that time: Skin hot and dry; tongue furred, white on centre, red at tip and edges; mouth dry and lips crusted; slight headache; pain in back and bowels; least pressure on abdomen causes pain; pulse 115, small and hard; dysuria for last twenty-four hours; has had two stools during same period from castor oil and spts. turpentine she had taken. *Diagnosis*—Incipient puerperal fever. *Prescription:* Pepper poultice to abdomen, warm cloths to vulva, spts. nitre in parsley tea every two hours, until dysuria is relieved.

5 o'clock, P. M. Has urinated tolerably freely since morning, less pain in bowels, pulse 120; other symptoms the same. *Prescription;* Eight drops tinct. veratrum viride every three hours until pulse is reduced.

8th—9 o'clock, A. M. Took but two doses of the veratrum viride, before nausea was induced, and lasted two to three hours. Simultaneously with the induction of nausea, perspiration set in, the pulse was lessened in frequency, and continued so all night. Present condition—Pulse 100, skin cool, tongue less red, feels but little pain and sleeps better, except during existence of nausea, than she had done in several nights. Took a dose of oil and turpentine at 1 o'clock this morning, which has not operated yet; feels nausea occasionally.

9 o'clock, P. M. One stool; feels better, nausea occasionally all day; pulse 85; has taken none of the veratrum viride since last night. Ordered 10 gtt. tr. veratrum viride every three hours, till nausea occurs, if fever returns. Has passed urine tolerably freely to-day.

8th. Has had no fever of consequence since last date, and therefore needed no medical attention.

Remarks.—This case would, in all probability, have terminated in puerperal fever, of which it presented the essential characters, but for the timely administration of hellebore. The febrile action could not have been owing to the dysuria and consequent distention of the bladder, for after these symptoms were relieved, the pulse numbered 120; neither could it be fairly attributed to want of action from the bowels, for they had been moved twice the day the first note was taken. She had had her "milk fever" three or four days before the date first noted, and got on well during the interim; the lochial discharge was normal, and the untoward features were attributed by the family to "a cold she had taken." It is reasonable, therefore, to infer that it would have run its course, as a case of puerperal fever, had not the potent agency of the veratrum viride curtailed its progress. This is the first recorded case of puerperal fever treated by the American hellebore, and the sanguine expectations of its worthy pioneer, Dr. Norwood, have been fully answered.

Case II. Nov. 8th, 1851.—Eliza, a negro woman, aged 25, strong and vigorous constitution, was taken with pneumonia of right lung on the 4th; was seen and treated by Dr. A. A. Bell until the 8th, after the usual manner. Calomel, tart. antimony, ipecac, Dov. powder and blisters, constituted the treatment. He had used the tinct. of veratrum viride freely for two days, without producing any perceptible effect whatever, in addition to the remedies above enumerated. The patient's condition at above date was as follows: Skin warm and moist, from ipecac and tart. antimony she had been taking freely all day; pulse 120–130, small, compressible under influence of tartar-emetic; bowels disposed to looseness, and tender to the touch. At 5 o'clock, P. M., she took 10 gtt. tinct. veratrum viride to control the circulation, which had not been influenced in the least by the calomel and tartar-emetic which she had been taking for twenty-four hours.

7 o'clock, P. M. Pulse the same; still perspiring—15 gtt. veratrum viride. 8 o'clock, P. M. Nausea and profuse perspiration; griping in bowels, which was followed by a copious watery operation; pulse 110—took 60 gtt. tinct. opii. to check bowels. 9 o'clock, P. M. Nausea and perspiration the same, bowels easy, feels drowsy, pulse 100, intermittent. 12 o'clock at night. Nausea and perspiration, pulse 100, not intermittent—took 10 gtt. veratrum viride.

9th—10 o'clock, A. M. One operation since last note, followed by 30 gtt. tr. opii; still perspires; skin cool; pulse 100, not intermittent; no pain; feels drowsy. Ordered, 10 gtt. veratrum viride every three hours while the pulse numbers 100—should it fall before that, to be given less frequently. Convalescence commenced at above date.

*Remarks.*—This case Dr. O'Keeffe saw in consultation with Dr. Bell, who had judiciously treated it with the usual remedies. He had even given the hellebore a fair and impartial trial for two successive days; but it failed —signally failed. The present case was the second in which the incomparable remedy, hellebore, failed in Dr. B.'s hands to produce any effect whatever, and he was on the eve of condemning it as an insignificant puff, when a different preparation proved to him satisfactorily that his was an inferior article—was inert. Should this disappointment happen to others, they would do well to pause, and consider the reliability and intelligence of their druggist. Dr. B. obtained his tinct. of veratrum viride from his druggist, while the preparation that fulfilled its mission—told the heart's arteries "so fast shalt thou beat and no faster," was prepared by a physician, in his own office, in the proportion of two ounces to the pint of alcohol.

It is difficult to say, with unerring certainty, what agency the veratrum had in bringing on a favorable crisis in this case, or how it would have terminated under the usual treatment. She took the first dose of hellebore at 5 o'clock, P. M., the tongue being moist and skin warm, but moist also; notwithstanding these favorable effects from the calomel and tart. emetic, the pulse was not reduced any. Two doses of the hellebore accomplished what several doses of the tart. antimony had failed to do. A crisis might have taken place without the veratrum, and its occurrenee, after its exhibition, may have been a coincidence instead of an effect; but from the infallible certainty with which it reduces the action of the heart and arteries, I think it not unreasonable to attribute the favorable change, in the present case, to its potency.

Case III. Mr. B., aged 45, complained of cold chills and acute pain in left side on 12th of November. Poultices, baths, diaphoretics and expectorants, failing to relieve him, he was bled next day 20 oz., the pulse being 100, full and strong. The v. s. and perspiration that followed relieved the pleuritic pain, partially, for a time, but it returned next day with some severity. A blister was applied to the affected side, which removed the pain; took 15 grs. of calomel, twice, in divided doses, which produced three dark operations. During this time, the tongue was covered on the middle with a whitish fur, natural at edges; the mouth dry, clammy, and exceedingly unpleasant, required cleansing frequently in the day; the pulse during the whole time 90–100.

8th day of illness. Skin hot, harsh and dry; tongue furred, clammy and sticking to roof of mouth; taste unnatural, very feeble and extremely restless; pain no where, pulse 90 to 100, variable, feeble and hard. Took several doses of diaphoretic preparations, until altering the condition of the skin, which was so hot and dry that he said he felt like he should "burn up." At 10½ o'clock, A. M., took 10 gtt. veratrum viride, the pulse being 100. At 11 o'clock, the skin was warmer and disposed to become moist; pulse stronger and more frequent, 114; no nausea. 1 o'clock, P. M. Skin moist, generally; large drops of sweat on face, drowsy, no nausea; pulse 114. 3 o'clock. Vomited considerable quantities of bilious matter several times. After he had vomited twice, skin became cool and dry, and the pulse fell to 100; and after he had vomited two or three times more, the pulse fell to 80, was small and weak. 5 o'clock. The vomiting having continued, and feeling griping pain in lower bowels, he took two tea-spoonfuls of paregoric, which checked the vomiting and griping. Expectoration, of which there was little or none before nausea, became profuse after it, and relieved the lungs very much. He took no more of the hellebore, and his pulse fell to 60, and continued so for several days, when convalescence commenced, and progressed satisfactorily to recovery.

*Remarks.*—Here is a case in which the usual diaphoretic remedies had been used freely and faithfully, without effecting the desired result; whereas 10 gtt. of the veratrum acted like a charm. The hellebore was not given in this instance to diminish the frequency of the pulse—for that was not at all imperative—but to act simply as a diaphoretic, and to remove the great heat of which the patient complained so much. It is proper to note that, before nausea set in, and after taking the veratrum, the temperature of the surface was heightened, and the pulse accelerated from 100 to 114 in the minute—a result that ensued only in the present instance.

Without further notice of this case, which was considered one of typhus fever, I propose to give, in detail, the notes of the case of pneumonia in which the hellebore was administered as a principal remedy during the whole progress of the disease.

Case IV. Nov. 23d, 1851, 9 o'clock, P. M. A negro girl, aged 20, of strong constitution, in seventh month of pregnancy, felt pain (slight) for the first time, in her left side, this morning, but attended to her usual duties until three hours before she was prescribed for. Condition: acute pain in her left side; cough hard, painful and frequent; skin hot and dry; tongue clean; pulse 120, and strong. *Diagnosis*—Pleuro-pneumonia.

*Prescription:* Venesection 1 quart; poultice to left side, and 10 gtt. tinct. veratrum viride. 12 o'clock. The same; 15 gtt. veratrum viride. 3 o'clock, A. M. The same; 20 gtt. veratrum viride.

24th, second day, 7 o'clock, A. M. Skin a little cool, vomited some since last note, perspired some during nausea; other symptoms the same—23 gtt. veratrum viride. 8 o'clock, A. M. Nausea and vomiting several times, skin natural, pain almost relieved; pulse 80, small and weak; deathly sick—Ginger tea to relieve nausea, and poultice to chest. 6 o'clock, P. M. Took the veratrum every three hours since last note; skin warm and dry; one stool; pain in side returned; pulse 112—15 gtt. veratrum viride. 8 o'clock. Vomited three times; skin moist; pain in side the same; pulse 80. ℞. Calomel, Dov. powder, *aa* 5 grs.; ipecac, 3 grs.; to be taken at once —cups over seat of pain, and poultice.

25th—9 o'clock, A. M. One stool; emesis several times in night; skin harsh and dry, but natural in temperature; cough hard and painful; pulse 120—15 gtt. veratrum viride every three hours, poultice to chest, and expectorant to relieve cough. 7 o'clock, P. M. Took three 15 gtt. doses of the hellebore, after the last of which she vomited three times; no stool; skin cool; pulse 80; respiration rapid, 50 in the minute. 11 o'clock, P. M. Took no medicine since last note; respiration rapid, embarrassed and difficult; skin cool, but harsh and dry; very restless, pulse 120, hard and constricted—15 gtt. veratrum viride in 50 gtt. tr. opii., to produce sleep.

26th—1½ o'clock, A. M. Took since last note 15 gtt. veratrum viride: respiration and pulse the same; skin warm and moist, is in a copious perspiration; no nausea —20 gtt. veratrum. 8 o'clock, A. M. Respiration and pulse the same; skin dry and warm; no stool; pulse 120 20 gtt. veratrum viride. 9 o'clock, A. M. Was sitting up when last note was made, which perhaps accelerated the pulse somewhat; skin a little moist and warm; pulse 100, lessened 20 beats without any nausea or vomiting; "felt worms creeping up her throat," attended with a flow of saliva and a desire to heave. (Complained of this sensation of worms in the throat since she has been taking the hellebore, and the same feeling was described by another patient who was taking the veratrum at the same time.) 15 gtt. veratrum viride every three hours, and intermediately a powder of calomel, Dover's powder and ipecac; blister to left side. 7½ o'clock, P. M. Took the above; no stool; after second dose of veratrum, vomited freely; skin warm; perspired none; blister well drawn; cough loose; pulse 90—continued veratrum every three hours, and gave injection to relieve bowels.

27th—9 o'clock, A. M. Two stools; emesis several times; cough very troublesome, causing vomiting; respiration very rapid, 50–60; pulse 100—15 gtt. veratrum viride, and same powders intermediately; blister dressed.

28th—8 o'clock, A. M. Followed prescription; two stools; constant nausea and vomiting; cough hacking and troublesome; tongue slightly furred; respiration same; pulse 100—continued hellebore and powders. Night visit: No improvement; felt labor pains since last note. *Prescription:* Discontinued hellebore; blisters to legs and right side; quinine, Dov. powder and ipecac, every three hours, and tart. antimony alternately.

29th—9 o'clock, A. M. Miscarried: pulse very frequent; getting worse—same treatment continued; brandy and quinine if pulse should sink. Night visit: Dead.

*Remarks.*—This case of pneumonia tested the hellebore to its utmost capacity, and brings home to our mind the very reasonable conviction that it will not prove infallible in the treatment of pneumonia. On reviewing these notes, it will be seen that the heart and arteries seldom failed to respond to the sedative influence of the veratrum, while the respiration was not affected by it in the least: it was a singular phenomenon to observe the respiration as frequent as 50 or 60, the skin cool and moist and the pulse 80. Hence, in this case, at least, it did control the circulation, but did not affect the respiration or inflammation. There is every reason to believe that the pneumonia inflammation received no check whatever, notwithstanding the circumstance that the patient was kept fully and constantly under the influence of the hellebore. But it is a question of reasonable doubt, whether any course of treatment would have averted the inevitable doom that awaited this patient in view of her advanced state of pregnancy. It is the duty of physicians who submit this agent to the test of clinical experience, to watch carefully its effects, and report them for the benefit of the profession: the remedy is in a state of probation preparatory to its admittance into the sanctum of practice, and nothing should be withheld that may add to its value or detract from its merits. In view of the severity and well marked character of the diseases in which it is most likely to be used, its effects can be early determined, either as positive or negative: it will control the bounding pulse, or it will not; there is no half-way ground—no room for speculative propensities. In a type of typhoid fever, in which pulmonic symptoms predominated, Dr. O'K. assures us it had the happiest effects in curtailing the duration of the fever and expediting convalescence. But in cases in which inflammation of the intestinal mucous membrane exist, its exhibition may prove somewhat questionable. It may be said, that as far as the cases go, the claims of the American hellebore to a potent remedy are founded on no fictitious representations: they rest on the indisputable facts of rigid experience; and we have little doubt that when it shall have passed through an extensive trial, the conviction of its importance will become universal, and in the language of Dr. Norwood, a new era in the treatment of disease will be the consequence.—*Southern Med. & Surg. Journal.*

